# Involvement of Calcium-Mediated Reactive Oxygen Species in Inductive GRP78 Expression by Geldanamycin in 9L Rat Brain Tumor Cells

**DOI:** 10.3390/ijms140919169

**Published:** 2013-09-18

**Authors:** Fang-Chun Sun, Hsin-Yi Shyu, Meng-Shiou Lee, Meng-Shiunn Lee, Yiu-Kay Lai

**Affiliations:** 1 Department of Bioresources, Da-Yeh University, Changhua 515, Taiwan; 2 Department of Life Science and Institute of Biotechnology, National Tsing Hua University, Hsinchu 300, Taiwan; E-Mails: a0523001@ms21.hinet.net (H.-Y.S.); yklai@life.nthu.edu.tw (Y.-K.L.); 3 School of Chinese Pharmaceutical Science and Chinese Medicine Resources, China Medical University, Taichung 404, Taiwan; E-Mail: leemengshiou@mail.cmu.edu.tw; 4 Department of Medical Research, Tungs’ Taichung MetroHarbor Hospital, Taichung 435, Taiwan; E-Mail: mengshiunn@yahoo.com.tw

**Keywords:** geldanamycin, calcium, PKC, ROS, GRP78

## Abstract

Treatment with geldanamycin (GA) leads to an increase in [Ca^2+^]_c_ and the production of reactive oxygen species (ROS) in rat brain tumor 9L RBT cells. GA-exerted calcium signaling was blocked by BAPTA/AM and EGTA. The effect of GA on [Ca^2+^]_c_ was significantly reduced in the presence of thapsigargin (TG) and ruthenium red (RR). GA-induced GRP78 expression is significantly decreased in the presence of BAPTA/AM, EGTA and RR, suggesting that the calcium influx from the extracellular space and intracellular calcium store oscillations are contributed to by the calcium mobilization and GRP78 expression induced by GA. The induced GRP78 expression is sensitive to added U73122 and Ro-31-8425, pinpointing the involvement of phospholipase C (PLC) and protein kinase C (PKC) in GA-induced endoplasmic reticulum (ER) stress. The antioxidants *N*-acetylcysteine (NAC), BAPTA/AM, EGTA and H7 also have significant inhibitory effects on ROS generation. Finally, neither H7 nor NAC was able to affect the calcium response elicited by GA. Our results suggest that the causal signaling cascade during GA-inducted GRP78 expression occurs via a pathway that connects PLC to cytoplasmic calcium increase, PKC activation and, then, finally, ROS generation. Our data provides new insights into the influence of GA on ER stress response in 9L RBT cells.

## 1. Introduction

Geldanamycin (GA), a naturally occurring benzoquinoid ansamycin antibiotic, has been identified as an agent that exhibits potent antitumor activity [1,2]. Several GA derivates have entered clinical trials as adjuvants to conventional chemotherapy [3,4]. GA specifically binds to the ATP-binding site of HSP90 and interferes with its function by inhibiting the protein’s ATPase activity. This causes the degradation of several key signaling proteins or, alternatively, the downregulation of their substrates; these are involved in gene expression, cell cycle progression and apoptosis [5,6]. Releasing of heat shock factor (HSF) from HSP90 complex activates the expression of heat shock proteins (HSPs) through heat shock response elements (HSEs) in their promoters [7].

GA is also a potent inducer of the endoplasmic reticulum (ER) stress response. GA, exerting its cellular effect by accumulation of mal-processed proteins in ER lumen, is shown by inductive expression of ER stress hallmark gene *grp78* [8]. HSP90 inhibition induces ER stress, which leads to disruption of mitochondrial homeostasis, leading to apoptosis. Induction of ER stress leads to upregulation of ER chaperones, GRP78 and GRP94, cleavage of caspase12 and an increase in cytoplasmic calcium [9]. Moreover, GA carries a benzoquinone moiety that generates ROS [10,11]. GA had been found to react non-enzymatically with glutathione (GSH), resulting in cellular GSH depleting [12].

Our previous study has shown that GA-induced *grp78* expression involves activation of ROS via ER stress responsive elements (ERSEs) in 9L RBT cells [13]. GA treatment is known to cause a transient increase in intracellular Ca^2+^ and transactivated HSP70-1/2 isoform expression in H460 cells through signaling pathways mediated by Ca^2+^ and PKC [14,15]. An increase of intracellular calcium, oxidative stress and ER stress are the effects of GA that have previously been reported, but the mechanism mediating these effects remain unclear and has not been directly addressed. Using GA-induced GRP78 expression as the end point assay, this work unraveled the causal relationships between calcium signaling and ROS generation by employing a battery of inhibitors targeting the major signaling mediators involved in the processes. In the study, we investigated and characterized the signaling pathways involved in GA-induced ER stress.

## 2. Results

**Figure 1:**
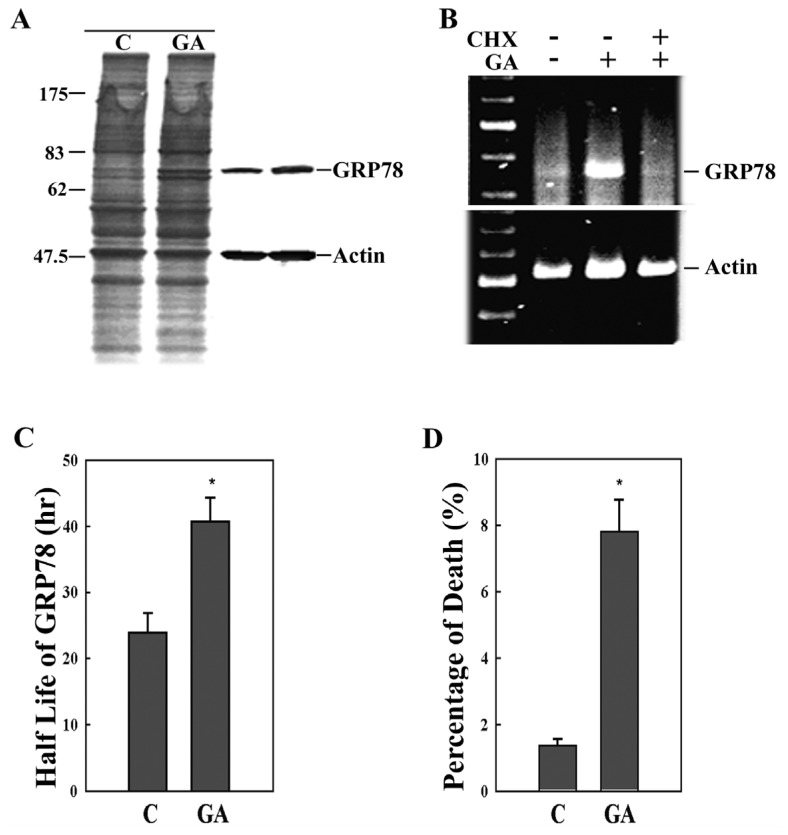
GA induces GRP78 expression and cell death. (**A**) The cells were exposed to solvent control (C) or 5 μM geldanamycin (GA) for 6 h and labeled with [^35^S] methionine for 1 h before being harvested. GRP78 proteins were analyzed by autoradiography (left panel) and Western blotting (right panel); (**B**) The cells were incubated with cycloheximide (CHX) for 1 h followed by GA treatment for 6 h. After treatment, total RNA was extracted and analyzed for the expression of *grp78* mRNA by RT-PCR; (**C**) Chase analysis of the sulfur incorporated into GRP78 in the absence or presence of GA. GRP78 half-life was determined from the logarithmic values of the chased time points compared to the unchased controls; (**D**) Cells were treated with DMSO (control) or 5 μM GA for 36 h. Cell death percentage was measured by trypan blue staining. Data represent the means ± SD of three independent experiments. The statistical significance of the differences were relative to control as measured by the *t*-est. *p < 0.05.

GA is a potent ER stress inducer that evidently upregulates *grp78* mRNA accumulation and GRP78 protein synthesis. This is confirmed in Figure 1, which shows cells treated with 5 μM GA for 6 h, where the GRP78 synthesis was monitored by metabolic labeling with [^35^S]-methionine. An increased rate of GRP78 synthesis can be observed (Figure 1A, left panel). A comparable upregulation in GRP78 protein was also confirmed by Western blotting (Figure 1A, right panel). The induced *grp78* mRNA expression is mainly on the transcriptional level, which was confirmed by the RT-PCR experiments; thus, the increase in translation of GRP78 caused by GA is largely due to a concomitant increase in the mRNA transcription. The induced *grp78* mRNA expression is completely abolished in the presence of cycloheximide (CHX) (Figure 1B), indicating that intact protein synthesis machinery is necessary for GA-mediated *grp78* induction. To determine whether the stability of GRP78 protein is modulated in GA-treated cells, a pulse label and chase analysis was performed. Control and GA-treated cells were labeled with [^35^S]-methionine for 30 min and chased in the common culture medium for various periods. It takes 24 h for the ^35^S- labeled GRP78 to be metabolize to 50% activity in the control cells, while GA-induced ^35^S-GRP78 persists significantly longer (Figure 1C). The presence of GA lengthens the half-life of GRP78 protein in 9L RBT cells. Experiments in the current study also showed that GA has anti-proliferative activity in 9L RBT cells (Figure 1D).

**Figure 2:**
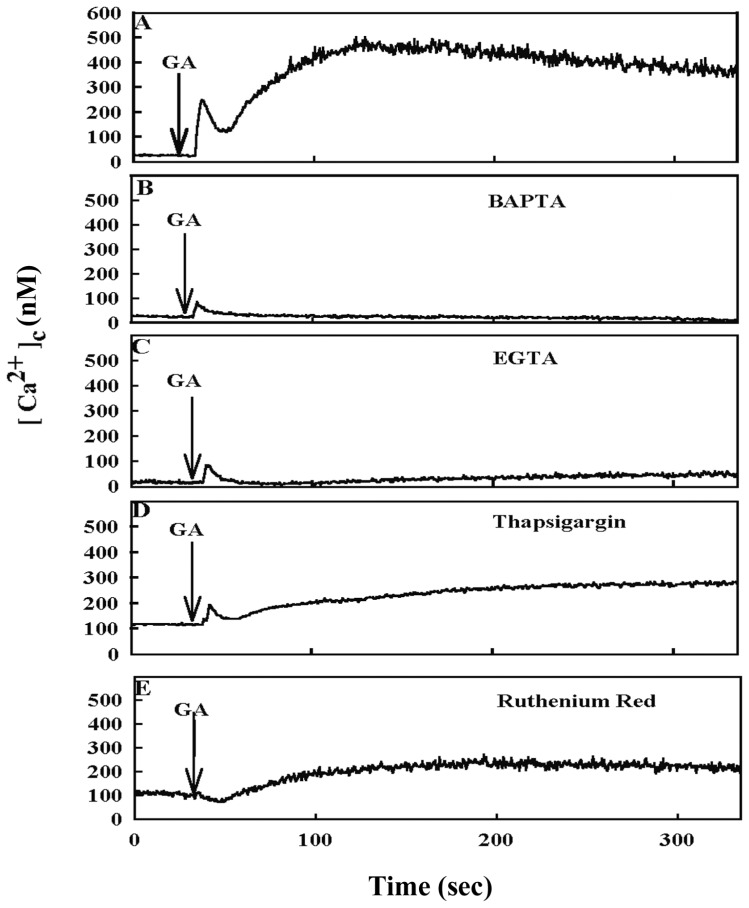
Effects of BAPTA/AM, EGTA, thapsigargin and ruthenium red on GA-induced intracellular calcium concentration. Cells were loaded with indo-1/AM. GA only as control (**A**) or preincubated with 15 μM BAPTA-AM (**B**); 1 mM EGTA (**C**); 300 nM thapsigargin (TG) (**D**); or 50 μM ruthenium red (RR) (**E**) for 20 min and, then, stimulated with GA at time 0. Changes in [Ca^2+^]_c_ were determined by fluorescence microphotospectrometry.

To investigate whether GA induces a rise in intracellular calcium in 9L RBT cells, experiments were conducted to monitor the changes in intracellular calcium using the calcium-sensitive dye, Indo-1/AM, by fluorescence microscopy. As shown in Figure 2, a remarkable increase in the intracellular calcium concentration occurred within seconds of GA treatment and was sustained for minutes after the addition of GA. GA evoked a rapid and two-phase increase in calcium, consisting of an initial spike of about 250 nM (phase I) within five seconds, which was followed by a slower subsequent response (phase II) that reached a higher level of about 450 nM; phase II lasted for several minutes or more (Figure 2A).

Membrane pumps in the ER, mitochondria and plasma membrane work in concert to maintain the intracellular calcium level. To address whether the GA-mediated cytoplasmic calcium signaling is derived from intracellular or extracellular calcium, the cells were pretreated with the intracellular calcium chelator (BAPTA-AM) or the extracellular calcium chelator (EGTA) and, then, treated with GA. The amplitude of phase I of calcium signaling was decreased, and phase II drops almost to baseline in the presence of BAPTA/AM and EGTA (Figure 2B,C). The contribution of intracellular calcium to the GA effect was determined by depleting intracellular calcium ER store with TG and by noncompetitive inhibition of the mitochondrial calcium uniporter with ruthenium red (RR). TG reduces the amplitudes of both phase I and phase II of calcium signaling, which are evoked by GA by about 50% (Figure 2D). RR completely abolishes phase I calcium signaling and reduces phase II signaling to about 50% (Figure 2E). These results suggest that the effect of GA on calcium signaling involves both extracellular and intracellular calcium sources.

**Figure 3:**
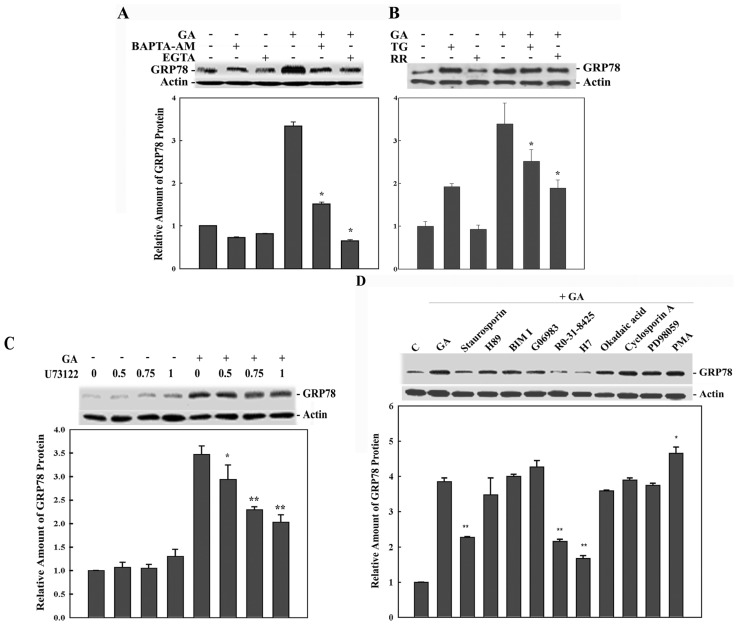
Involvements of calcium, PLC and PKC in GA-induced GRP78 expression. Cells were pre-incubated for 1 h with BAPTA-AM, EGTA (**A**); TG or RR (**B**) and followed by GA treatment for 6 h. Whole cell lysate was subjected to Western blotting. β-actin served as a loading control. The data represent the means ± SD of three independent experiments; *p < 0.01, compared with GA treatment only; (**C**) Cells were exposed to various concentrations of U73122 for 1 h and, then, treated with GA for 6 h; (**D**) cells were incubated for 1 h with 1 μM staurosporin, 10 nM H89, 20 nM bisindolylmaleimide I, 10 μM Go6983, 20 μM Ro-31-8425, 40 μM H7, 60 nM okadaic acid, 50 nM cyclosporin A, 50 μM PD98059 or 1 μM phorbol 12-myristate 13-acetate and followed by GA treatment for 6 h in the presence of the drugs. The samples were processed for Western blotting, followed by quantitative analysis. Bar graphs represent the means ± SD of three independent experiments; *p < 0.05, **p < 0.01, compared with the treatment with GA only.

To determine whether the GA-induced GRP78 and the rise of [Ca^2+^]_c_ are casually related, we examined the effect of calcium chelators and calcium pump inhibitors on the enhanced expression of GRP78 in GA-treated cells. BAPTA-AM reduces the GA-induction of GRP78 expression to 45%, and EGTA reduces the GA-induction of GRP78 to 21% (Figure 3A). Inhibition of the mitochondrial calcium uniporter by RR and SERCA by TG diminish the GA-induced GRP78 expression to 55% and 77%, respectively (Figure 3B). These results indicate that calcium mobilization from both intracellular stores and extracellular influx is involved in GA-induced GRP78 expression. The influx of calcium into cells might affect a second messenger through activation of a protein phosphorylation cascade. Protein kinase C (PKC) is activated in a canonical fashion by diacyl glycerol (DAG) and Ca^2+^ [16]. It was likely that GA may activate phospholipase C (PLC), which would lead to the production of DAG and inositol 1,4,5-triphosphate (IP_3_). U73122, which inhibits PLC, was found to decrease GA-induced GRP78 expression in a concentration-dependent manner (Figure 3C), suggesting that GA may stimulate PKC activation via activation of PLC, which then causes the [Ca^2+^]_c_ elevation.

PKC has many isoenzymes classified into three groups: calcium- and diacylglycerol (DAG)-dependent classical PKC (cPKC-α, -βI, -βII and -γ), calcium-independent and DAG-dependent novel PKC (nPKC-δ, -ɛ, -η, -θ and -μ), and calcium- and DAG-independent atypical PKC (aPKC-ξ and -ι) [17]. When 9L RBT cells were treated with general protein kinase inhibitor staurosporine and nonspecific PKC inhibitor H7, the GRP78 induction was significantly inhibited. Ro-31-8425, a selective PKC inhibitor [18], was able to inhibit the inductive effect on GRP78 by GA. An enhancement in GRP78 expression was also found after pretreatment with the PKC activator, phorbol 12-myristate 13-acetate (PMA) (Figure 3D). Data obtained with these inhibitors employed in this study can only suggest an implication of PKC and PLC involved in GA-induced GRP78 expression. However, the subtype of PKC or PLC needs more specific strategies to be examined. In addition, no appreciable changes were found after pretreatment with H89, okadaic acid (OA), cyclosporin A (CsA) and PD98059, which implies that protein kinase A, protein phosphatase 1, protein phosphatase 2A, protein phosphatase 2B and MEK1/2 can be excluded from involvement as major signal transduction conduits in GA-induced GRP78 expression (Figure 3D).

**Figure 4:**
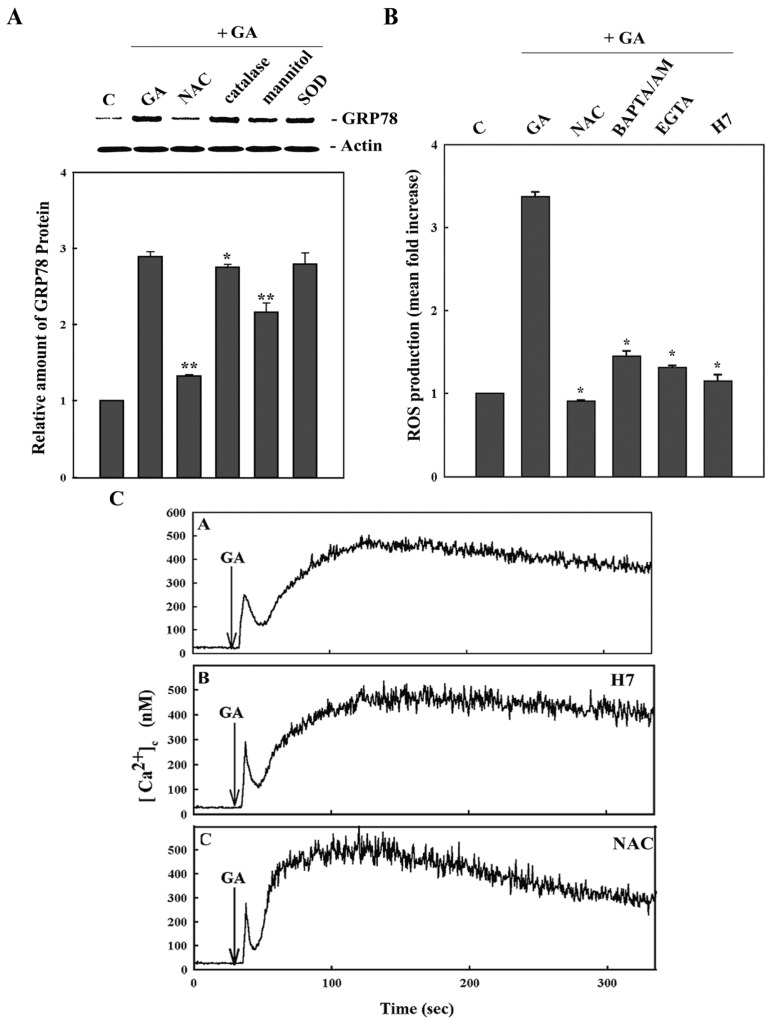
The effects of GA involve calcium, protein kinase C and the reactive oxygen species pathways. (**A**) Cells were, respectively, incubated for 1 h with 5 mM *N*-acetylcysteine (NAC), 50 units/mL catalase, 100 mM mannitol or 100 units/mL superoxide dismutase (SOD) and followed by GA treatment for 6 h. The GRP78 and β-actin level were analyzed using Western blotting. Data represent the means ± SD of three independent experiments, and the statistical significance of the differences were relative to GA only, as measured by the *t*-test; *p < 0.05, **p < 0.01; (**B**) Cells were incubated for 1 h with 5 mM NAC, 15 μM BAPTA-AM, 1 mM EGTA or 40 μM H7, respectively, and followed by GA treatment for 1.25 h before the determination of the ROS level. The data represent the means ± SD of three independent experiments, and the statistical significance of the differences were relative to GA only, as measured by the *t*-test; *p < 0.01; (**C**) Cells were loaded with indo-1/AM and, then, preincubated with 40 μM H7 (middle panel) or 5 mM NAC (lower panel) for 20 min, then stimulated with GA; GA only was used as a control (upper panel). Changes in [Ca^2+^]_c_ were determined by fluorescence microphotospectrometry. The results were obtained from three separate experiments.

An oxygen molecule generates intermediate species, including superoxide radical (O_2_^−^), hydrogen peroxide (H_2_O_2_) and hydroxyl radicals (OH^−^). The intermediates, superoxide radical and hydroxyl radical, are important free radicals that cause peroxidation of intracellular lipids and tissue damage. A superoxide radical is converted by superoxide dismutase (SOD) to hydrogen peroxide, which is eventually converted to water by catalase. Superoxide radical is converted by the Fenton reaction and Haber-Weiss reaction to a hydroxyl radical [19,20]. In order to identify the different ROS involved in GA-induced GRP78 induction, several ROS scavengers, such as mannitol (a hydroxyl radical scavenger), superoxide dismutase (SOD, catalyzing the degradation of O_2_^−^), catalase (catalyzing auto-oxidoreduction of H_2_O_2_) and *N*-acetylcysteine (NAC, the precursor of glutathione), were employed to examine the individual inhibitory effect. Catalase had little effect on the GRP78 expression induced by GA. Mannitol is more effective at inhibiting ROS than the others in directing GA signaling (Figure 4A). Our results indicate that GA induced ROS production, and hydroxyl radical is the prominent chemical species of ROS involved in GA-induced GRP78 expression. However, the inhibitory effect of the general ROS scavenger, NAC, is much more remarkable than mannitol. This suggests that these ROS inducing GRP78 expression may be compensatory and not dependent on individual special chemical species only.

To determine the causal relationship between ROS production and calcium signaling, we studied the effects of calcium chelators, ROS scavengers and PKC inhibitors on the signaling pathways. A significant increase in ROS production was detected in cells treated with GA only. When the cells were pretreated with NAC, BAPTA-AM, EGTA and H7 (a non-specific PKC inhibitor), the production of ROS induced by GA was effectively prevented (Figure 4B). This suggests that the intracellular calcium concentration modulates ROS homeostasis. Notably, BAPTA-AM and EGTA could not suppress ROS production to the basal level compared with control cells, suggesting another calcium-independent signal pathway to make ROS in GA-treated 9L RBT cells. In combination with calcium monitoring, the presence of NAC or H7 results in the suppression of ROS generation, but these inhibitors were unable to modulate the calcium signaling evoked by GA (Figure 4C). These results support the idea that calcium entry and protein kinase signaling act upstream of ROS generation. Indeed, the present study showed that GA causes ROS generation mostly through a Ca^2+^-dependent mechanism. The underlying mechanism by which the intracellular calcium rise stimulates ROS generation remains to be defined.

## 3. Discussion

Tight regulation of organelle calcium mobilization and GA-induced GRP78 expression are revealed. In the present study, GA evoked a rapid and two-phase increase in calcium, consisting of an initial spike within five seconds, which was followed by a slower subsequent response and lasted for several minutes in 9L RBT cells (Figure 2A). The inducted expression of GRP78 in GA-treated cells can be effectively abolished by the calcium-modulating agents, EGTA and BAPTA-AM (Figure 3A), under which the GA-induced increase in [Ca^2+^]_c_ is also significantly prevented (Figure 2B,C). The general mechanisms of calcium homeostasis are common to all eukaryotic cells. Extracellular Ca^2+^ can cross the plasma membrane via voltage-operated Ca^2+^ channels (VOCCs) and receptor-operated channels (ROCs) [21]. Stimulation of ryanodine receptors (RyRs) and IP_3_ receptors in the ER may increase the intracellular calcium level [22]. In the present study, the plasma membrane does comes into play, as evidenced by the fact that EGTA is able to block the GA-induced calcium influx and that inhibition of PLC by U73122 reduces GRP78 induction by GA (Figures 2C and 3C). Removal of extracellular calcium almost completely abolished the calcium elevation and the expression of GRP78 induced by GA (Figures 2C and 3A), implying that GA-induced GRP78 expression is largely due to an influx of extracellular calcium through the plasma membrane. However, PLC may be involved in the mitochondrial calcium handling. Mitochondrial calcium uptake is sensitive to U73122 in a concentration-dependent manner [23]. Based on this, the effects of PLC inhibition on intracellular calcium signaling may also involve changes in mitochondrial calcium handing. The rise in intracellular calcium level and GRP78 induction were partially suppressed by treatment with TG and RR, which suggests that calcium mobilization in ER and mitochondria is also involved in the GA effect (Figures 2D,E and 3B). Nonetheless, more experiments are warranted to further investigate the direct effect of GA on calcium influx and the involvement of intracellular calcium stores.

An elevated level of [Ca^2+^]_c_ can lead to excessive ROS production, while excessive ROS production can lead to cytosolic Ca^2+^ overload [24]. Organelle calcium mobilization may simply be involved in alleviating the accumulation of a lethal load of calcium by mitochondria and ER buffering in order to prevent adverse oxidative stress and cell death. There are multiple sources of ROS in the cell, including nicotinamide adenine dinucleotide phosphate (NADPH) oxidase (NOX), xanthine oxidase (XO), uncoupling of nitric oxide synthase (NOS), cytochrome P450 and the mitochondrial electron transport chain (ETC) [25]. Generation of mitochondrial ROS (mtROS) takes place at the ETC located on the inner mitochondrial membrane during the process of oxidative phosphorylation. Both superoxide and hydrogen peroxide in this process are considered as mtROS. Endogenous modulators, such as NO and Ca^2+^, can regulate the production of mtROS by regulating the metabolic states of mitochondria [26]. Moreover, mitochondria also participate in Ca^2+^ homeostasis. Mitochondrial Ca^2+^ overload increases mtROS production, which is independent of the metabolic states of mitochondria [27]. Under certain conditions, non-mitochondrial-generated ROS can enhance mtROS production [28,29]. There is a reciprocal relationship between many of these sources of ROS. For example, astrocytes produce ROS through NOX [30]. ROS produced by NOX can alter mitochondrial function and increase mtROS production [28].

NOX enzymes may be activated through Ca^2+^ signaling, but the regulation of Ca^2+^ signaling through NOX also occurs. NOX enzymes may regulate the activity of the plasma membrane Ca^2+^ channels, the intracellular Ca^2+^ release channel and Ca^2+^ pumps through ROS-dependent posttranslational modification and electron transport-dependent cell depolarization [31]. The relationship between ROS and Ca^2+^ regulation is complicated and an interesting intracellular regulatory system. The derangement of this precisely controlled system would lead to the development of many diseases. According to previous study, GA was shown to increase the generation of superoxide, which scavenges and reduces NO bioavailability through the flavin-containing enzyme cytochrome P-450 reductase, bypassing HSP90, in epithelial cells and vascular smooth muscle cells [11]. In *Sf9* cells, GA is capable of producing hydrogen peroxide by neuronal nitric oxide synthase (nNOS), and the flavin domain of NOS may mediate the electron transfer reactions for redox cycling of GA [32,33]. Hydrogen peroxide is shown to interact with intracellular iron to produce highly reactive hydroxyl radicals. Furthermore, GA cytotoxicity involves disruption of HSP90 binding and requires oxidative stress, but does not require proteasomal or lysosomal activity to induce protein degradation and cell death [34]. GA and its analogs are capable of participating in redox cycling by redox activation through the semiquinone intermediate to generate reactive oxygen species, which account for the oxidative stress [35]. More investigation needs to be conducted to clarify whether GA-induced ROS generation in 9L RBT cells comes from nNOS or NOX.

Inhibition of HSP90 by GA leads to ER stress-induced mitochondrial-mediated apoptosis, and it is well known that Bax and calcium play an important role in mitochondrial damage [9]. Induction of ER stress proteins may prevent a general disturbance of intracellular calcium homeostasis by alleviating the accumulation of the unfolded proteins [36]. GRP78 has been shown to prolong cell survival and to block the apoptosis that is caused by alterations in intracellular calcium [37]. GRP78 may stabilize mitochondrial permeability and attenuate ER stress-induced apoptosis by maintaining Raf-1 stability and activity [38]. Overexpressing GRP78 protects astrocytes against ischemic injury, reduces net flux of Ca^2+^ from ER to mitochondria, increases Ca^2+^ uptake capacity in isolated mitochondria, reduces radical production and preserves respiratory activity and mitochondrial membrane potential after stress [39]. In the present study, the presence of GA lengthens the half-life of the GRP78 protein, which may be an adaptation and prosurvival response (Figure 1C). The conditioning 9L RBT cells with GA increased expression of GRP78, while causing cell death after a long time treatment (Figure 1D). Organelle calcium and ROS influence *grp78* induction, and these might also influence the oncoprotein Bcl-2, which is lodged both in the mitochondria and the ER, and have an effect on cell survival [40]. The balance between anti-apoptotic and pro-apoptotic molecules, such as Bcl-2 and Bax, respectively, is necessary for maintaining the homeostasis between cell survival and death. Bcl-2 overexpression reduces ER Ca^2+^ content and confers resistance against apoptosis [41]. Moreover, Bax and Bak function to elevate the resting ER Ca^2+^ concentration and to trigger the efflux of the ER Ca^2+^ into the cytosol and the uptake of the Ca^2+^ by mitochondria during severe ER stress [9,42,43].

GA treatment leads to increased intracellular calcium levels and ROS production and activates ER stress. GRP78, as a major ER molecular chaperone, may provide a buffer against GA-induced increase in cytosolic Ca^2+^ levels, hence suppressing apoptosis. The upregulation of the chaperone machinery of the cell could be promoting cancer cell survival and proliferation [44,45]. Elevated levels of GRP78 are frequently documented in tumor tissue, where this protein contributes to cellular survival and resistance against chemotherapy [46,47]. Therefore, downregulation of GRP78 may become an important adjunct in treatments of cancers. GRP78 is regarded as an ER stress marker and draws attention to linking ER stress to brain disorders [48,49]. Many neurodegenerative processes are associated with change in calcium homeostasis and ROS production. It has been reported that GRP78 is expressed at low levels in normal adult brain, but is significantly elevated in malignant glioma specimens and human malignant glioma cell lines, correlating with their rate of proliferation. The combination of drugs capable of suppressing GRP78 with a conventional agent might represent a novel approach to eliminate residual tumor cells after surgery, increasing the effectiveness of chemotherapy [45]. Treatment with geldanamycin (GA) leads to an increase in [Ca^2+^]_c_, the production of reactive oxygen species (ROS) and GRP78 induction in rat brain tumor cells. Calcium mobilization and ROS production induced by GA might reveal a neuronal, survival or death significance.

This is the first report to address the causative roles of calcium signaling, protein kinase C and ROS in GA-induced GRP78 expression. The present study showed that GA causes ROS generation mostly through a Ca^2+^-dependent mechanism. Our results suggest that the causal signaling cascade during GA-inducted GRP78 expression occurs via a pathway that connects PLC to cytoplasmic calcium increase, PKC activation and, then, finally, ROS generation. The data provides new insights into the influence of GA on ER stress in 9L RBT cells and may lead to the development of novel therapies for the treatment of cancers and neurological disorders.

## 4. Experimental Section

### 4.1. Cell Cultures and Drug Treatments

The 9L RBT cells [50] were maintained in Eagle’s minimum essential medium plus 10% fetal bovine serum supplement with 100 units/mL penicillin G and 100 μg/mL streptomycin at 37 °C under 5% CO_2_ and 95% air. Exponentially growing cells at 80%–90% confluence were used. To investigate the effects of GA, cells were treated with 5 μM GA for 6 h at 37 °C. For studies concerning the effect of inhibitors, the cells were preincubated with the various kinase inhibitors at the specified concentrations for 1 h before subjecting them to a range of treatments, as described in the figure legends.

### 4.2. Metabolic Labeling and SDS-PAGE

The *de novo* protein synthesis rate was measured by [^35^S] methionine labeling at a concentration of 10 μCi/mL. After various treatments, the cells were labeled for 1 h, washed twice with PBS and lysed in 2× sample buffer. Equal amounts of cell lysates were resolved by the standard SDS-PAGE technique. After electrophoresis, the gels were fixed, dried and processed for autoradiography using X-ray films. Protein bands of interest were quantified by densitometric scanning (Molecular Dynamics, Inc., Sunnyvale, CA, USA).

### 4.3. Pulse-Chase Analysis of GRP78

Cells were cultured in 60-mm dishes until at about 80% confluency and, then, were incubated for 20 min in methionine-free RPMI 1640 medium supplemented with 2 mM glutamine and 10% fetal bovine serum. Next, they were pulse-labeled for 30 min using 0.1 mCi of [^35^S]-methionine. To chase the ^35^S-labelled proteins, the cells were washed two times with PBS and placed in fresh complete medium for the indicated periods. Finally, the lysates were subjected to SDS-PAGE analysis. For the GA-treated samples, 5 μM GA was added to the methionine-free medium, retained throughout the labeling period and included in the chase medium. Densitometric scanning was performed to quantify the protein signals (Molecular Dynamics, Inc., Sunnyvale, CA, USA).

### 4.4. Western Blotting

The resolved proteins were transferred to nitrocellulose membrane (Hybond C; Amersham Int., Amersham, Little Chalfont, UK). The membrane was incubated with a 1:1000 dilution of anti-GRP78 (Affinity BioReagents, Golden, CO, USA) or a 1:1000 dilution of anti-β-actin (Sigma, St. Louis, MO, USA) at 4 °C for 2 h. After washing with PBS, the membrane was incubated with 1:1000 dilutions of horseradish peroxidase-conjugated anti-rabbit or 1:2000 of anti-goat secondary antibodies at 4 °C for 2 h. The immunocomplexes were detected using the enhanced chemiluminescence detection system (Amersham, Arlington Heights, IL, USA).

### 4.5. Measurement of Intracellular Free Calcium

Cells cultured on coverslips in 6-well plates were incubated with the calcium probe, indo-1/AM, in darkness at 25 °C for 45 min prior to the experiments in which [Ca^2+^]_c_ was monitored by single-cell dual-wavelength microfluorometry (PhoCal Pro., Life Science Resources, Cambridge, UK). The indo-1-loaded cells were illuminated at an excitation wavelength of 340 nm. The fluorescent intensities at emission wavelength of 405 nm (Ca^2+^-bound form) and 490 nm (free form) were measured simultaneously by two photomultipliers and were integrated for 100-ms intervals. The concentration of intracellular free calcium was estimated from the ratio, *R*, of the two emitted fluorescence intensities according to the following equation: [Ca^2+^]_c_ = *K*_d_ × (*R* − *R*_min_)/(*R*_max_ − *R*) × (*S*_f2_/*S*_b2_), where *R*_min_ is the limiting value of the ratio, *R*, when all the indicators are in the Ca^2+^-free form and *R*_max_ is the limiting value of *R* when the indicator is saturated with calcium. Experimentally, the factor, *S*_f2_/*S*_b2_, is simply the ratio of the fluorescence intensity measured when all the indicators are free (*S_f2_*) to intensity measured when all the indicators are Ca^2+^ bound (*S*_b2_); both measurements are taken at 490 nm. The *K*_d_ value of 250 nM had been determined previously *in vitro* [51]. For data presentation, at least three cells were chosen per experiment, and the changes in ratio corresponding to indo-1 fluorescence were calculated for each cell.

### 4.6. Determination of ROS Production

For the detection of intracellular ROS production in ER-stressed cells, we measured the reduction of the redox dye, nitroblue tetrazolium (NBT) (Sigma, Saint Quentin Fallavier, France). Cells were washed, and 100 μL of various solutions, namely PBS control, NBT only or NBT with GA, were added per well and incubated for 1.25 h in the dark at room temperature. The medium was removed, and cells were fixed in 70% methanol (Sigma, Saint Quentin Fallavier, France) and allowed to air dry. The formazan in each well was then dissolved in 2M KOH (Sigma, Saint Quentin Fallavier, France) and DMSO. The turquoise blue-colored solution was then read using a spectrophotometer at the wavelength, 630 nm [52].

### 4.7. Statistics Analysis

Data presented the means ± SD of three independent experiments. The statistical significance of the differences was relative to the control, as measured by the *t*-test. *p* < 0.05 was considered significant.

## 5. Conclusions

**Figure 5:**
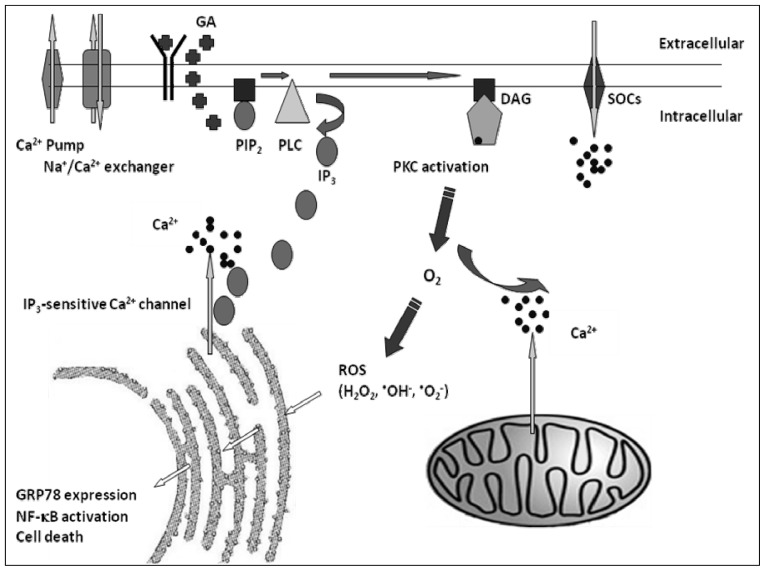
Model of GRP78 induction by calcium, PKC and ROS, following GA stimulation. DAG, diacyl glycerol; IP_3_, inositol 1,4,5-triphosphate.

Based on the present findings, we have proposed a model of the GA-induced ER stress signaling pathways in 9L RBT cells (Figure 5).
